# Increased hypothalamic serotonin turnover in inflammation-induced anorexia

**DOI:** 10.1186/s12868-016-0260-0

**Published:** 2016-05-20

**Authors:** J. T. Dwarkasing, R. F. Witkamp, M. V. Boekschoten, M. C. Ter Laak, M. S. Heins, K. van Norren

**Affiliations:** Nutrition and Pharmacology Group, Division of Human Nutrition, Wageningen University, Bomenweg 2, 6703HD Wageningen, The Netherlands; Nutrition, Metabolism and Genomics Group, Division of Human Nutrition, Wageningen University, Bomenweg 2, 6703HD Wageningen, The Netherlands; Brains On-line, P.O. Box 4030, 9701 EA Groningen, The Netherlands

## Abstract

**Background:**

Anorexia can occur as a serious complication of disease. Increasing evidence suggests that inflammation plays a major role, along with a hypothalamic dysregulation characterized by locally elevated serotonin levels. The present study was undertaken to further explore the connections between peripheral inflammation, anorexia and hypothalamic serotonin metabolism and signaling pathways. First, we investigated the response of two hypothalamic neuronal cell lines to TNFα, IL-6 and LPS. Next, we studied transcriptomic changes and serotonergic activity in the hypothalamus of mice after intraperitoneal injection with TNFα, IL-6 or a combination of TNFα and IL-6.

**Results:**

In vitro, we showed that hypothalamic neurons responded to inflammatory mediators by releasing cytokines. This inflammatory response was associated with an increased serotonin release. Mice injected with TNFα and IL-6 showed decreased food intake, associated with altered expression of inflammation-related genes in the hypothalamus. In addition, hypothalamic serotonin turnover showed to be elevated in treated mice.

**Conclusions:**

Overall, our results underline that peripheral inflammation reaches the hypothalamus where it affects hypothalamic serotoninergic metabolism. These hypothalamic changes in serotonin pathways are associated with decreased food intake, providing evidence for a role of serotonin in inflammation-induced anorexia.

**Electronic supplementary material:**

The online version of this article (doi:10.1186/s12868-016-0260-0) contains supplementary material, which is available to authorized users.

## Background

Loss of appetite (anorexia) leading to insufficient food intake is often seen in chronic illnesses including cancer, HIV and COPD. A chronically elevated increased inflammatory tone is considered one of the major drivers of anorexia in these diseases. Studies suggest that an ongoing elevated inflammatory tone in the hypothalamus, displaying the highest density of various cytokine receptors in the brain [1], is implicated in these disturbances in food intake. Inflammatory mediators affect important orexigenic and anorexigenic regulators including NPY [2] and POMC [3, 4] peptides in the hypothalamus. Cytokines and other pro-inflammatory signalling molecules from the periphery are able to reach the hypothalamus passing the blood brain barrier (BBB) [5–7]. In addition, de novo synthesis of various cytokines in the hypothalamus has been reported [8]. To trigger these processes, sensing of peripheral signals in the adjacent median eminence [9, 10] and activation of hypothalamic microglial and astrocyte cells [11, 12] might be crucial.

**Fig. 1:**
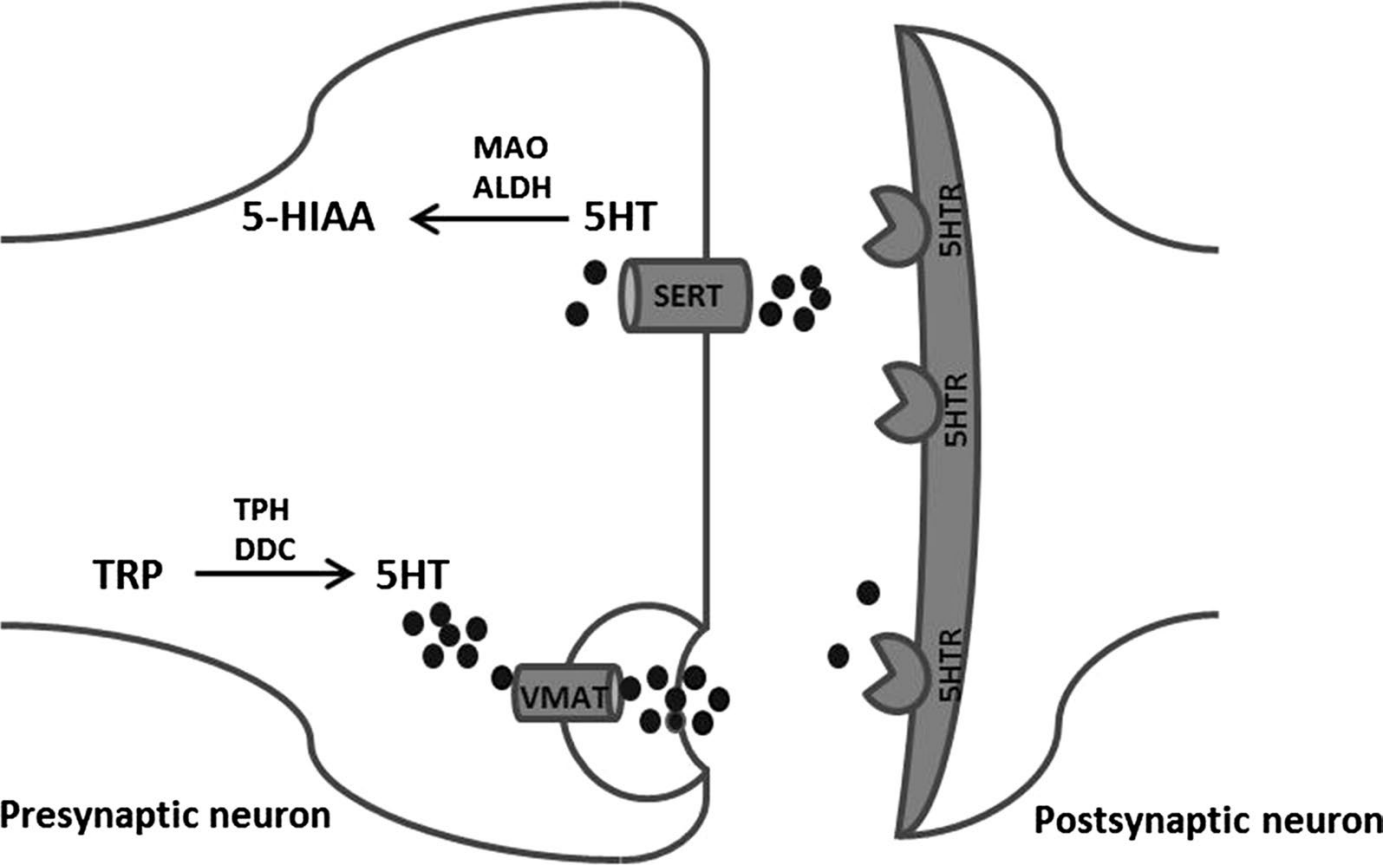
Serotonergic transmission in neurons. Conversion of tryptophan (TRP) to serotonin (5HT) is catalysed by TPH and DDC. Serotonin is then, by transportation via VMAT, stored into vesicles before it can be released into the synaptic cleft. There, serotonin signalling resulting from binding to serotonin receptors (5HTR) can be terminated by the reuptake of serotonin via SERT transporter. Once serotonin is taken up into the presynaptic neuron it is degraded by MAO and ALDH to 5-HIAA, which is considered a marker for serotonergic activity and measurable for a longer period compared to levels of secreted serotonin. *TRP* tryptophan, *TPH* tryptophan hydroxylase, *5-HT* serotonin, *5HTR* serotonin receptor, *5-HIAA* 5 hydroxyindolacetic acid, *DDC* dopadecarboxylase, *VMAT* vesicular monoamine transporter, *SERT* serotonin reuptake transporter, *MAO* monoamine oxidase, *ALDH* aldehyde dehydrogenase

We previously described changes in hypothalamic serotonin signalling in rodent tumour models displaying severe body wasting (cachexia). These changes in serotonin formation were inversely associated with food intake [13]. This is in line with findings in a variety of chronic illnesses, where increased hypothalamic serotonin has been implicated for its role in the development of disease-associated anorexia [14–17]. Hypothalamic serotonin plays an important role in food intake, since it is able to respond to peripheral signals on energy status [18–20] and it is able to modulate anorexigenic and orexigenic signalling in the hypothalamus. Serotonin is able to affect food intake via activation of the anorexigenic melanocortin system involving 5HT2c receptors [21] and by inhibition of the orexigenic NPYergic system [15, 22]. Furthermore, reduction of brain serotonin by reducing availability of its precursor tryptophan (TRP) (Fig. 1) has been shown to be beneficial in the treatment in anorexia during cancer [23].

In the present study, we investigated the anorexigenic effects of TNFα and IL-6, cytokines that are often elevated during chronic illness, on hypothalamic serotonin signalling. To test physiologically relevant concentrations of TNFα and IL-6 in illness, we included two combinations containing both TNFα and IL-6 that reflected plasma levels measured in C26 adenocarcinoma tumour-bearing mice or Lewis Lung tumour-bearing mice respectively [13, 24]. We show that these cytokines when administered intraperitoneally (ip) induce changes in the hypothalamic transcriptome consistent with changes in inflammatory pathways and serotonin signalling. Furthermore, these cytokines alter hypothalamic levels of serotonin’s main metabolite 5-HIAA (Fig. 1), indicating that synaptic serotonin release [25, 26] and serotonin turnover [27, 28] is affected by inflammation.

## Results

### IL-6, TNFα and LPS increase inflammatory markers, 5HT and 5-HIAA in hypothalamic cell lines

**Fig. 2:**
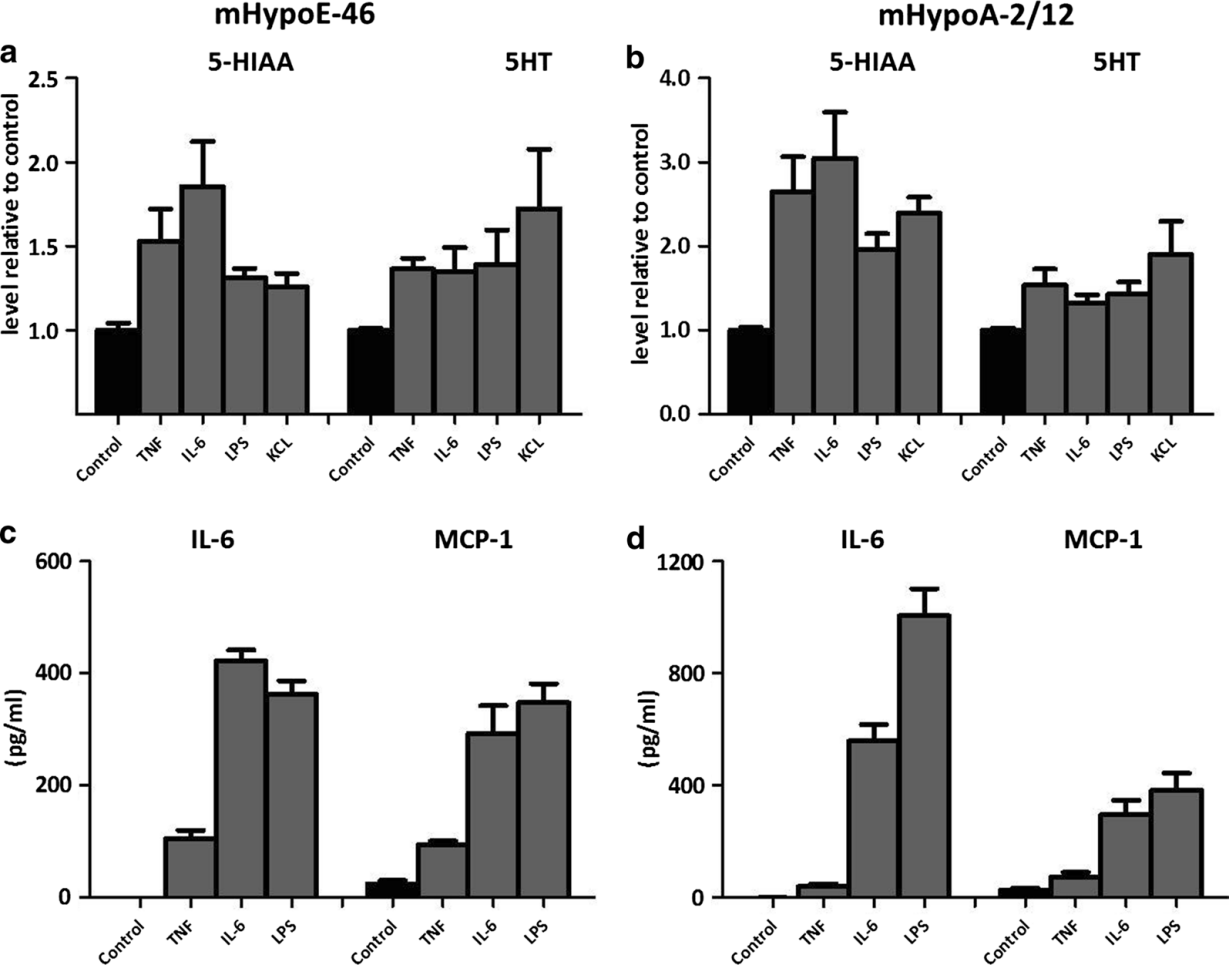
Effect of TNFα, IL-6 and LPS on 5-HIAA, 5HT, IL-6 and MCP-1 in HypoE-46 and mHypoA-2/12 cells. Murine derived hypothalamic cell lines were 24 h exposed to various concentrations TNFα (100 pg/ml), IL-6 (100 pg/ml) and LPS (1 μg/ml). KCL was used to depolarize cells (positive control for 5HT). **a**, **b** Intracellular 5-HIAA and secretion of 5HT in HypoE-46 (**a**) and HypoA-2/12 cells (**b**). **c**, **d** Production of IL-6 and MCP-1 in HypoE-46 (**c**) and HypoA-2/12 cells (**d**). All treatments were significantly different from untreated controls. Data are expressed as mean ± SEM (n = 3)

Exposure of hypothalamic cell lines hypoE-46 and hypoA2/12 to IL-6 (100 pg/ml), TNFα (100 pg/ml) and LPS (1 μg/ml) for 24 h stimulated serotonin (5HT) release into the medium. Furthermore, levels of intracellular 5-hydroxyindoleacetic acid (5-HIAA) were elevated after exposure to IL-6, TNFα and LPS in both cell lines. Both cell lines produced IL-6 when exposed to IL-6, since levels detected were four to sixfold higher than exposed levels. In addition, both cell lines produced MCP-1 when exposed to LPS and IL-6. Compared to IL-6 and LPS, TNFα showed to be less potent in inducing the production of MCP-1 and IL-6 (Fig. 2). No TNFα release was detected (data not shown) after exposure to IL-6, TNFα or LPS.

### IL-6 and TNFα reduce food intake in mice

**Fig. 3:**
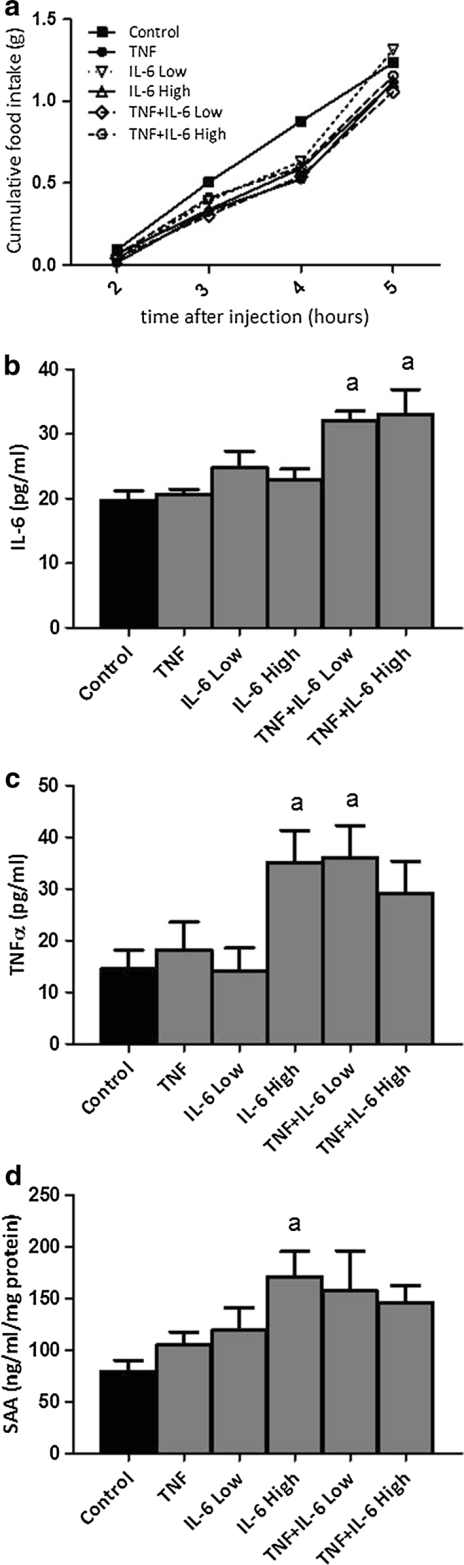
Effect of injection with TNFα, IL-6 or both on food intake and plasma cytokines. **a** Time course of food intake after injection with TNFα, IL-6 or both. **b**, **c** IL-6 and TNFα plasma levels 5 h after injection. **d** Level of serum amyloid 1 (SAA) in liver homogenates 5 h after injection. ^a^Significantly different from Control group (P < 0.05). Data is expressed as mean ± SEM (n = 12)

Hourly food intake remained constant in control animals during the entire study period. In cytokine treated mice, food intake curves started to deviate from 2 h after injection, becoming significantly lower 4 h after injection in the TNFα, IL-6 high, IL-6 Low + TNFα and IL-6 High + TNFα groups, compared to controls (Fig. 3). These effects did not differ between cytokine treatments. After 5 h of injection, food intake between all groups was similar again.

Plasma levels of cytokines and serum amyloid A levels in liver were measured 5 h after injection. There were three groups with significant changes in plasma TNFα, IL-6 or liver SAA levels: the two combination groups and the IL-6 high group. Plasma IL-6 was significantly higher in the two combination groups, while TNFα was increased in the IL-6 high and the TNF + IL-6 Low group. The elevation of the other combination group did not reach significance. Liver SAA was only significantly increased in the IL-6 high group (Fig. 3). Plasma levels of MCP-1, leptin, resistin, PYY, amylin, GIP, GLP-1 and insulin were not different between groups (Additional file 1: Figure 1). Ghrelin, pancreatic peptide and glucagon plasma levels were below detection limit of the assay.

### IL-6 and TNFα increase hypothalamic 5-HIAA and TRP

**Fig. 4:**
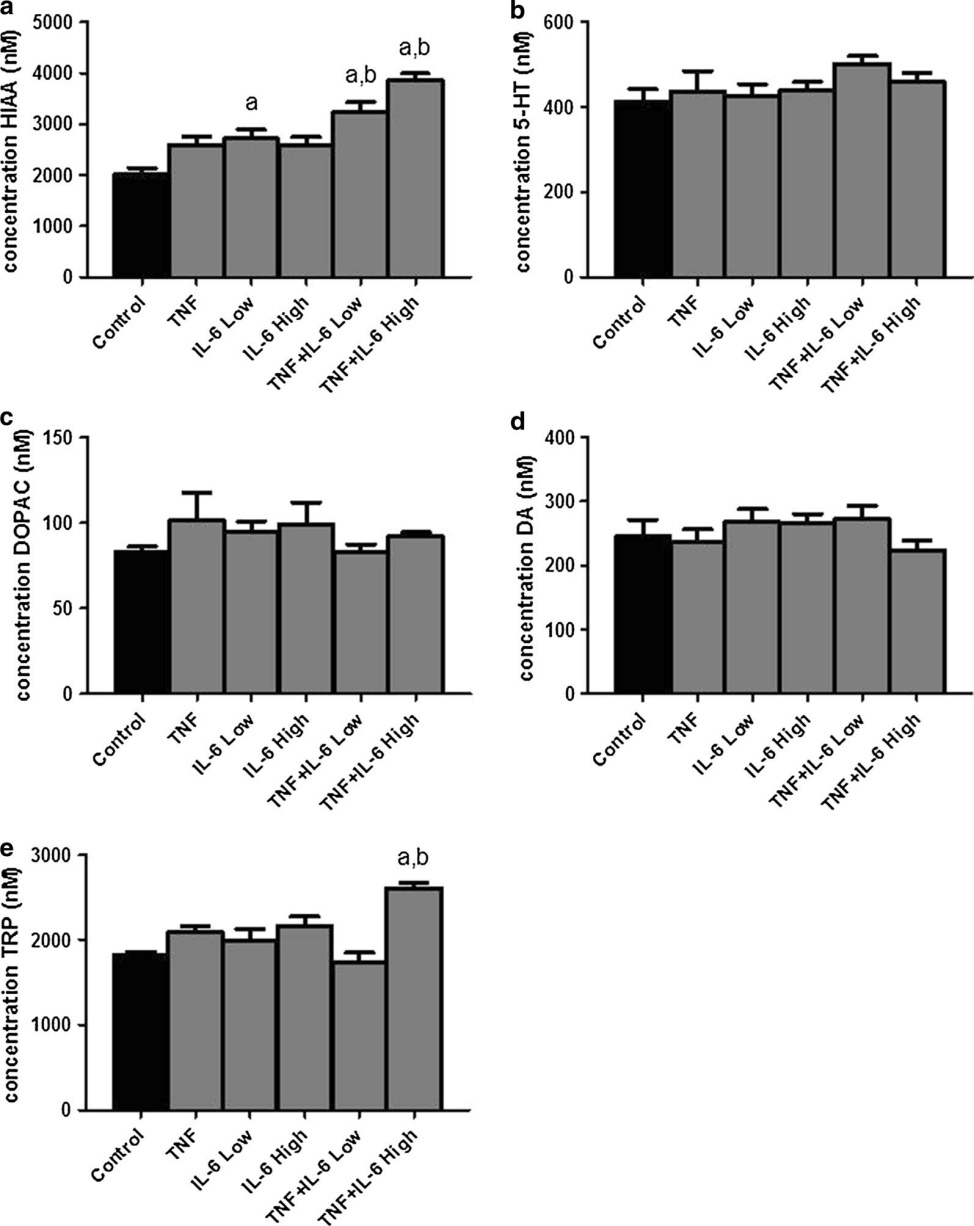
Levels of tryptophan, serotonin and dopamine and their metabolites in the hypothalamus. Effect of ip injection with TNFα, IL-6 or both on **a** 5-hydroxyindoleacetic acid (5HIAA), **b** Serotonin (5HT), **c** 3,4-dihydroxyphenylacetic acid (DOPAC), **d** Dopamine (DA) and **e** tryptophan (TRP). ^a^Significantly different from Control group (P < 0.05), ^b^significantly different from TNF, IL-6 Low and IL-6 High group (P < 0.05). Data is expressed as mean ± SEM (n = 6)

Total hypothalamic serotonin (5HT) tissue concentrations were not affected by injection with TNF and/or IL-6 5 h after injection. However, serotonin’s metabolite, 5-hydroxyindoleacetic acid (5HIAA), showed to be significantly elevated in hypothalamus homogenates of both the TNF + IL-6 Low and TNF + IL-6 High groups compared to controls (Fig. 4). These increases were more prominent than those following injection with TNF, IL-6 Low and IL-6 High alone. Tryptophan (TRP) showed to be significantly higher in mice injected with TNF + IL-6 High compared to controls. Hypothalamic levels of dopamine (DA) and its metabolite 3,4-dihydroxyphenylacetic acid (DOPAC) showed no differences between groups (Fig. 4).

### Hypothalamic transcriptome analysis: IL-6 and TNFα have similar effects on serotonin signalling and inflammatory pathways

**Fig. 5:**
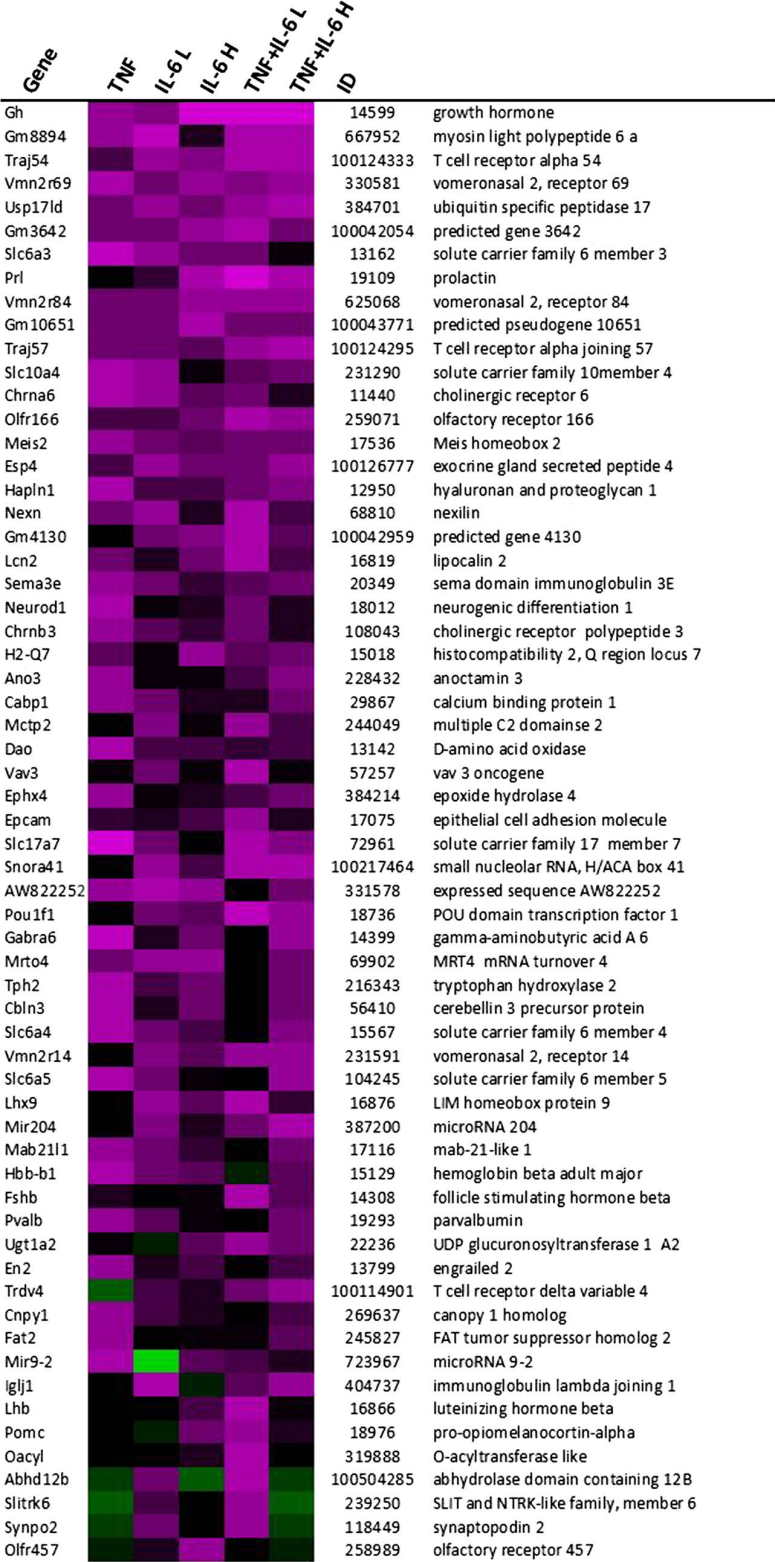
Gene expression changes in hypothalamus after ip injection with TNFα, IL-6 or both. Top upregulated genes and top downregulated genes in treated groups compared to control group. *Each row* represents a gene and *each column* represents a group of animals. *Magenta colour* indicates genes that were higher expressed as control and *green colour* indicates genes that were lower expressed as the control. *Black* indicates genes whose expression was similar to compared to control. ID: Entrez ID

**Fig. 6:**
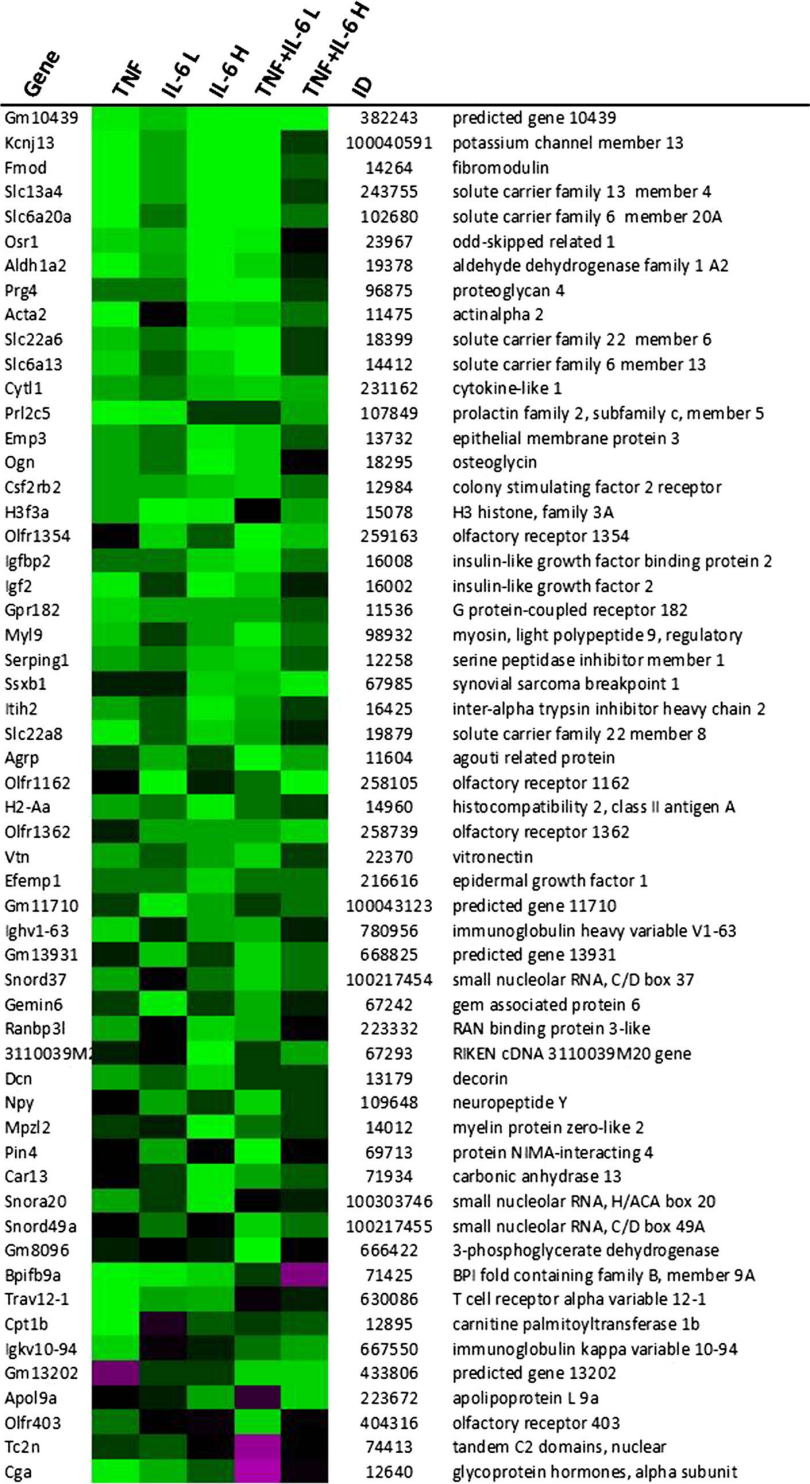
Gene expression changes in hypothalamus after ip injection with TNFα, IL-6 or both. Top upregulated genes and top downregulated genes in treated groups compared to control group. *Each row* represents a gene and *each column* represents a group of animals. *Magenta colour* indicates genes that were higher expressed as control and *green colour* indicates genes that were lower expressed as the control. *Black* indicates genes whose expression was similar to compared to control. ID: Entrez ID

**Fig. 7:**
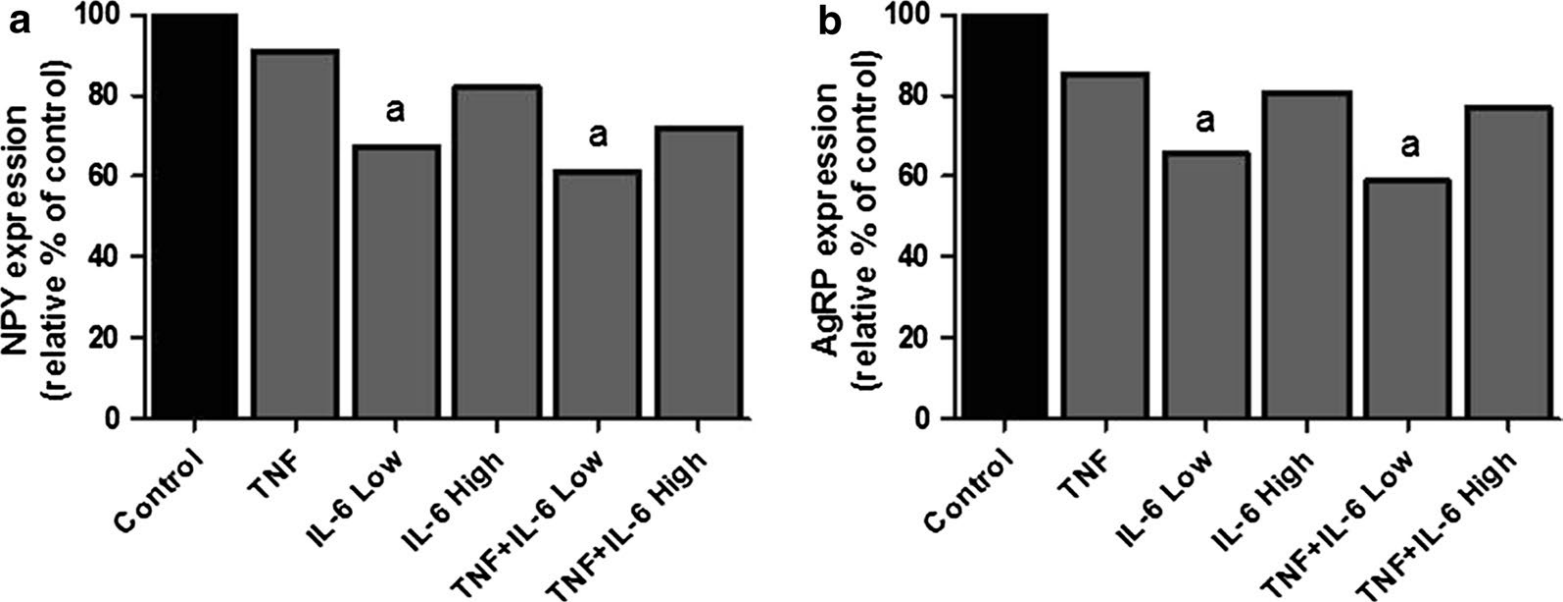
Gene expression changes in hypothalamus after ip injection with TNFα, IL-6 or both. **a**, **b** Relative gene expression compared to control group of NPY and AgRP. ^a^Significantly different from Control group (P < 0.05), *AgRP* agouti related protein, *NPY* neuropeptide Y (n = 6)

Expression of genes that were changed with a fold change greater than 1.5 compared to controls were compared among the different groups, resulting in a list of 118 genes (Figs. 5, 6, 7). From these genes, 87 genes showed to be altered in a similar direction (either up or down compared to controls) in at least 4 out of 5 treatment groups. Furthermore, 96 out of 118 genes were overlapping between animals from the TNF and IL-6 Low or IL-6 High groups. Altogether this shows that induced changes on gene expression were overall similar for treatment with TNF, IL-6 or the combination. More importantly, gene expression of rate-limiting enzymes involved in the synthesis of 5HT, including *Tph2* and expression of the serotonin-reuptake transporter, *Slc6a4*, were found to be among these highly upregulated genes. Strongly down-regulated genes included those of two important orexigenic regulators NPY and AgRP. Treatment effects showed to be similar for NPY and AgRP and these were most prominent in the IL-6 Low and TNFα + IL-6 Low groups (Figs. 5, 6, 7). Using Ingenuity, upstream regulators that were present in at least 3 treatment groups were listed (Table 1). Overall, this revealed an inflammatory profile of upstream regulators, which included cytokines IFNγ, TGFβ and IL-6 and the enzyme IKBKG which is an encoded protein of the IκB complex, and crucial for activating NFκB. The Ingenuity database used included 75 IFNγ target genes of which 19 out of 27 had an overlap with IL-6 target genes, which might explain the mutual presence of both these cytokines.

**Table 1:** Upstream regulators in hypothalamus after ip injection with TNFα and IL-6

Upstream regulators					
	TNF	IL-6 low	IL-6 high	TNF + IL-6 low	TNF + IL-6 high
Cytokines			IFNG	IFNG	
	IFNG	IFNG	IL6	IL6	
	IL6	IL6	PRL	PRL	IFNG
	TGFB1	PRL	TGFB1	TGFB1	TGFB1
Enzymes			TGFBR1	TGFBR1	
		TGFBR1	IKBKG	IKBKG	
	TGFBR1	IKBKG	ALDH1A2	ALDH1A2	
	ALDH1A2	PARP9	PARP9	PARP9	TGFBR1
Transcription factors			SRF		
			MKL1	SRF	
	SRF		SMAD7	MKL1	
	MKL1	SMAD7	CEBPB	CEBPB	SRF
	STAT1	CEBPB	STAT1	STAT1	MKL1
	IRF3	IRF3	IRF3	IRF3	SMAD7

## Discussion

Decreased food intake (anorexia) often occurs during conditions characterized by an elevated inflammatory response. In this study we investigated the role of serotonin in this decreased food intake during inflammation. Here, we show that both in vitro and in vivo hypothalamic inflammation is associated with increased serotonergic activity. Overall supporting the viewpoint that changes in hypothalamic 5HT signaling are involved in anorexia resulting from inflammation.

Interestingly, we found that the two hypothalamic cell lines used here (HypoE-46 and HypoA-2/12), which are derived from different neuronal populations of the hypothalamus, the PVN [29] and the ARC [30] respectively, are able to produce inflammatory mediators when exposed to LPS, TNFα or IL-6. Upon exposure to IL-6 and TNFα, neurons responded by the production of IL-6 and MCP-1, but not TNFα. MCP-1 production is regulated by IL-6, suggesting that MCP-1 production upon exposure to TNF might also involve the production of IL-6 [31].

So far, studies on hypothalamic inflammation have primarily focussed on the role of microglial and astrocyte activation [11, 12, 32, 33]. However, it has been shown before that neurons also express high levels of various cytokines [34] and that synthesis of cytokines occurs in response to blood-borne inflammatory mediators [35]. This suggests that neurons may play a significant role in development and sustainment of hypothalamic inflammation. Furthermore, we showed that this inflammatory response in hypothalamic neurons coincided with elevated serotonin secretion and increased intracellular 5-HIAA levels, reflecting an increase in serotonin turnover. In the mice, injection with combinations of TNFα and IL-6 resulted in increased hypothalamic 5-HIAA levels, while tissue serotonin levels did not differ from controls. A possible explanation for this apparent difference could be that in cell experiments, secreted serotonin was measured. However, in total tissue homogenates, intracellular serotonin cannot be distinguished from serotonin released into the synaptic cleft. Therefore, total tissue homogenate levels of serotonin do not properly reflect its release, which has also been reported for other monoamines and catecholamines [36]. However, 5-HIAA, the stable metabolic end product of 5HT is considered to be a good reflection for serotonin release [37–39] and therefore widely acknowledged as marker for serotonergic activity [25, 26].

Levels of 5-HIAA are measured in different matrices, models and diseases, including various forms of depression and aggressive behaviour disorders, where measurement of 5-HIAA gives better results than that of serotonin. Alterations on 5-HIAA are more pronounced and long-lasting, while changes on serotonin can be rapidly diminished [27, 40–42].

In the current experimental setting, 5 HT and metabolites were measured 5 h after cytokine injection and it could be that alterations of serotonin levels had already diminished by then. A similar reasoning might explain why elevated levels of serotonin’s precursor, tryptophan (TRP), were only prominent in mice injected with the combination of the highest dose of IL-6 and TNFα. Tryptophan levels have been reported to rise upon injection with cytokines depending on the type of inflammatory stimulus used. However at the same time, this inflammatory response is associated with increased TRP breakdown by indoleamine 2,3-dioxygenase (IDO), to synthesize kynurenine [43, 44].

In plasma, levels of TNFα and IL-6 were not elevated in the groups treated with a single cytokine. However, when these cytokines were given in combination, high levels of IL-6 and TNFα were measured. It is likely that clearance of the administered cytokines already occurred within 5 h following injection. For both TNFα and IL-6, complete clearance from the circulation has been reported to occur within 6 h after ip injection [45, 46]. Furthermore levels of these cytokines have different temporal profiles, with TNFα plasma being fast responsive and also more rapidly cleared than IL-6 from the blood, but at the same time being more persistent in hypothalamus than IL-6 [47, 48].

Food intake showed to decrease in all treated groups and there was no difference between the treatments. It appears that the lowest dose of IL-6 was sufficient to induce an anorexigenic effect and that higher doses produced no additional anorexigenic activity. This is in line with other reports showing that in contrast to other IL-6 effects, there is no clear dose-dependency of IL-6 when it comes to its activity on food-intake [49, 50].

Whole genome gene expression profiles from the hypothalamus and the predicted inflammatory transcriptional regulators showed a high overlap between TNFα, IL-6 and combination groups, indicating that responses of the different treatments were similar. This might be explained by a production of similar cytokines by the host in response to injection with IL-6 or TNFα. In hypothalamic tissue homogenates, increased levels of IL-6 have been reported after injection with TNFα [51], which suggests that injection with TNFα also leads to activation of IL-6 signalling pathways. The strong down-regulation of expression of two important orexigenic neuropeptide regulators NPY and AgRP also showed to be similar between groups and corresponded to lower food intake in treated groups. This decrease in expression induced by TNFα or IL-6 supports findings that have been reported in a variety of experimental models including hypothalamic injection with TNFα and genetic overexpression of hypothalamic IL-6 [52, 53].

Gene expression of two genes involved in serotonin signalling, showed to be upregulated in mice treated with TNFα and IL-6. Serotonin has been reported to have an inhibiting action on NPY [13, 54, 55], suggesting that the increase in serotonergic activity measured in treated mice, might be responsible for the effects on lower NPY expression in these mice.

## Conclusions

In summary, we show that TNFα and IL-6 induce similar inflammatory responses in the hypothalamus and similar effects on food intake. This anorexigenic effect of TNFα and IL-6 showed to coincidence with increased hypothalamic serotonergic activity, providing further evidence for a role for serotonin in inflammation-induced anorexia.

## Methods

### Cell culture and in vitro studies with IL-6, TNFα and LPS

Murine derived hypothalamic neuronal cell lines hypoE-46 and hypoA2/12 (CELLutions Biosystems Inc. Canada) were grown and maintained in DMEM supplemented with 10 % heat-inactivated fetal calf serum, 100 U/ml penicillin and 100 μg/ml streptomycin at 37 °C under 5.0 % CO_2_. Cells were grown in monolayers to 90 % confluency. Then medium was replaced by serum-free DMEM containing penicillin and streptomycin. After 4 h, cells were exposed to LPS (1 μg/ml), TNFα (100 pg/ml), IL-6 (100 pg/ml) for 24 h, or KCl (60 mM) for 15 min. After exposure, supernatant was collected to measure levels of serotonin (BAE-5900, LDN, Nordhorn, Germany), IL-6 (DY406, Abingdon, UK), TNFα (DY410, Abingdon, UK) and MCP-1 (DY479, R&D systems, Abingdon, UK) by enzyme-immuno assay. Cells were homogenized in 40 mM Tris, 1 mM EDTA, 5 mM EGTA and 0.50 % Triton X-100. Homogenates were used to measure 5-hydroxyindoleacetic acid (5-HIAA) by ELISA (MBS261481, MyBiosource, Breda, The Netherlands) and corrected for total protein content (Pierce Bicinchoninic acid Rockford, IL, USA). Cytotoxicity was determined by measuring LDH leakage and cell viability using an XTT conversion assay after 48 h of exposure (Roche Diagnostics, Mannheim, Germany). All experiments were performed three times in quadruplicate.

### Animals

C57BL/6 male mice (Harlan, Horst, The Netherlands), weighing approximately 20 g, were individually housed 1 week before start of the experiment. Mice were maintained on a 12 h light:12 h dark cycle in a climate-controlled room (21 ± 1 °C). Standard diet was ad libitum available during the entire experiment from one hour prior to dark phase until start of the light phase (Arie Blok B.V., Woerden, and The Netherlands). Water was freely available 24 h a day. Food intake, water intake and body weight were monitored daily from 1 week prior to the end of the experiment.

All experimental procedures were made in accordance with the European Community guidelines for the use of laboratory animals and complied with the principles of good laboratory animal care.

### Experimental set-up

Mice were injected intraperitoneal (ip) with 50ul of saline vehicle (G-Biosciences, St. Louis, USA), TNFα (Peprotech, London, UK), IL-6 (Peprotech, London, UK), or both TNFα and IL-6. The study included 6 groups: Control, TNFα, IL-6 Low, IL-6 High, TNFα + IL-6 Low and TNFα + IL-6 High (Table 2). The rationale to study different doses and combinations of IL-6 was based on our previous observations in mouse tumour models and the generally recognized central role of IL-6 as link between cancer and inflammation [13, 24]. Combinations TNF + IL-6 Low and TNF + IL-6 High reflect plasma levels measured in C26 tumour-bearing mice and Lewis Lung tumour-bearing mice respectively. Each group included 12 mice, of which 6 mice were used for determination of hypothalamic metabolites and 6 mice were used for hypothalamic gene expression analysis. Mice were injected 1 h prior to the dark phase. Five hours after injection, blood was collected by cardiac puncture under general anaesthesia. After sacrifice, brain, hypothalamus and organs were weighted, frozen in liquid nitrogen and stored at −80 °C.

**Table 2:** Experimental groups

Group	Nr of mice	Treatment (IP injection 50 μl sodium chlorine)
Control	12	Sodium chloride 0.9 %
TNF	12	15 pg TNFα
IL-6 low	12	50 pg IL-6
IL-6 high	12	800 pg IL-6
TNF + IL-6 low	12	50 pg IL-6 + 15 pg TNFα
TNF + IL-6 high	12	800 pg IL-6 + 15 pg TNFα

### Hypothalamic metabolites 5-HT, 5-HIAA, DA, DOPAC and TRP

Hypothalamus tissue was homogenised by sonication in 10 μl of 0.5 M perchloric acid per mg of tissue and stored at −80 °C until analysis. Concentrations of DA, 5-HT, DOPAC, 5-HIAA and TRP were determined by HPLC with tandem mass spectrometry (MS/MS) detection, using deuterated internal standards of the analytes. Of each LC–MS sample, an aliquot was injected onto the HPLC column by an automated sample injector (SIL10-20AC-HT, Shimadzu, Japan). Chromatographic separation was performed on a SynergiMax column (100 × 3.0 mm, particle size 3 μm) held at a temperature of 35 °C. The mobile phases consisted of A: ultrapurified H_2_O + 0.1 % formic and B: acetonitrile: ultrapurified H2O (75:25) + 0.1 % formic acid. Elution of the compounds proceeded using a suitable linear gradient at a flow rate of 0.3 ml/min. The MS analyses were performed using an API 4000 MS/MS system consisting of an API 4000 MS/MS detector and a Turbo Ion Spray interface (Applied Biosystems, the Netherlands). The acquisitions on API 4000 were performed in positive ionization mode for 5-HT, DA and TRP and in negative mode for 5-HIAA and DOPAC, with optimized settings for the analytes. The instrument was operated in multiple-reaction-monitoring (MRM) mode.

Data were calibrated and quantified using the Analyst data system (Applied Biosystems, version 1.6.2, the Netherlands). Concentrations in experimental samples were calculated based on the calibration curve in the corresponding matrix.

### Hypothalamic transcriptomics (microarray)

Total RNA from the hypothalamus was isolated by using RNeasy Lipid tissue kit (Qiagen, Venlo, The Netherlands). RNA concentrations were measured by absorbance at 260 nm (Nanodrop). RNA quality was checked using the RNA 6000 Nano assay on the Agilent 2100 Bioanalyzer (Agilent Techologies, Amsterdam, The Netherlands) according to the manufacturer’s protocol. For each mouse, total RNA (100 ng) was labelled using the Ambion WT expression kit (Life Technologies, Bleiswijk, The Netherlands). Micro-array experiments were performed by using Affymetrix Mouse Gene 1.1 ST arrays. In the TNFα treated group, 1 sample gave multiple spots on the array and was therefore excluded from analysis. Array data were analysed using an in-house, on-line system [56]. Briefly, probesets were redefined according to Dai et al. [57] using remapped CDF version 18.0.1 based on the Entrez Gene database. In total these arrays target 21,266 unique genes. Robust multi-array (RMA) analysis was used to obtain expression values [58, 59]. We only took genes into account that had an intensity >20 on at least 3 arrays and at least 7 probes per genes. Genes were considered differentially expressed at P < 0.05 after intensity-based moderated t-statistics [60]. Further functional interpretation of the data was performed through the use of IPA (Ingenuity^®^ Systems, www.ingenuity.com). Genes from the data set that met the cut-off of 1.2 fold change and P value cut-off of 0.05 were considered for the analysis. Upstream regulators were identified by using cut-off values of z-score >1.96 and z-score <−1.96 combined with P < 0.05. Furthermore for this upstream regulators analysis, only endogenous metabolites were considered (chemical drugs and compounds were excluded from analysis). Array data have been submitted to the Gene Expression Omnibus (GEO), accession number GSE69151.

### Plasma cytokines and gut hormones

Plasma levels of TNF-α, amylin (Active), C-Peptide 2, ghrelin (Active), GIP (Total), GLP-1 (Active), glucagon, IL-6, insulin, leptin, MCP-1, Pancreatic Peptide (PP), PYY and resistin were measured using the 12-plex Mouse Metabolic Hormone Magnetic bead panel (Merck Millipore, Amsterdam, The Netherlands). Serum amyloid was measured in liver homogenates using SAA mouse ELISA kit (Life technologies, Bleiswijk, The Netherlands).

### Statistics

Data were analysed by statistical analysis of variance (ANOVA) followed by a post hoc Bonferroni test or by a Dunnet test. Differences were considered significant at a two-tailed P < 0.05. Statistical analyses were performed using Graphpad Prism 5.

## Additional file

10.1186/s12868-016-0260-0 Plasma levels of gut hormones. Effect of ipinjection with TNFα, IL-6 or both on A) Resistin, B) Peptide YY, C) Glucagon–like peptide (GLP-1), D) MCP-1, E) Insulin, F) C-peptide, G) Gastric inhibitoryprotein (GIP), H) Amylin, I) Leptin.

## Abbreviations

5HIAA: 5-hydroxyindoleacetic acid
5HT: 5-hydroxytryptamine (serotonin)
AgRP: agouti related protein
BBB: blood brain barrier
IDO: indoleamine 2,3-dioxygenase (IDO)
IL-6: interleukin-6
NPY: neuropeptide Y
POMC: pro-opiomelanocortin
TNFa: tumor necrosis factor alpha
