# Supplementary figures and images for: Increased hypothalamic serotonin turnover in inflammation-induced anorexia
Source: BMC Neurosci. 2016 May 20;17:26. doi: 10.1186/s12868-016-0260-0 (PMC4875640; doi:10.1186/s12868-016-0260-0)

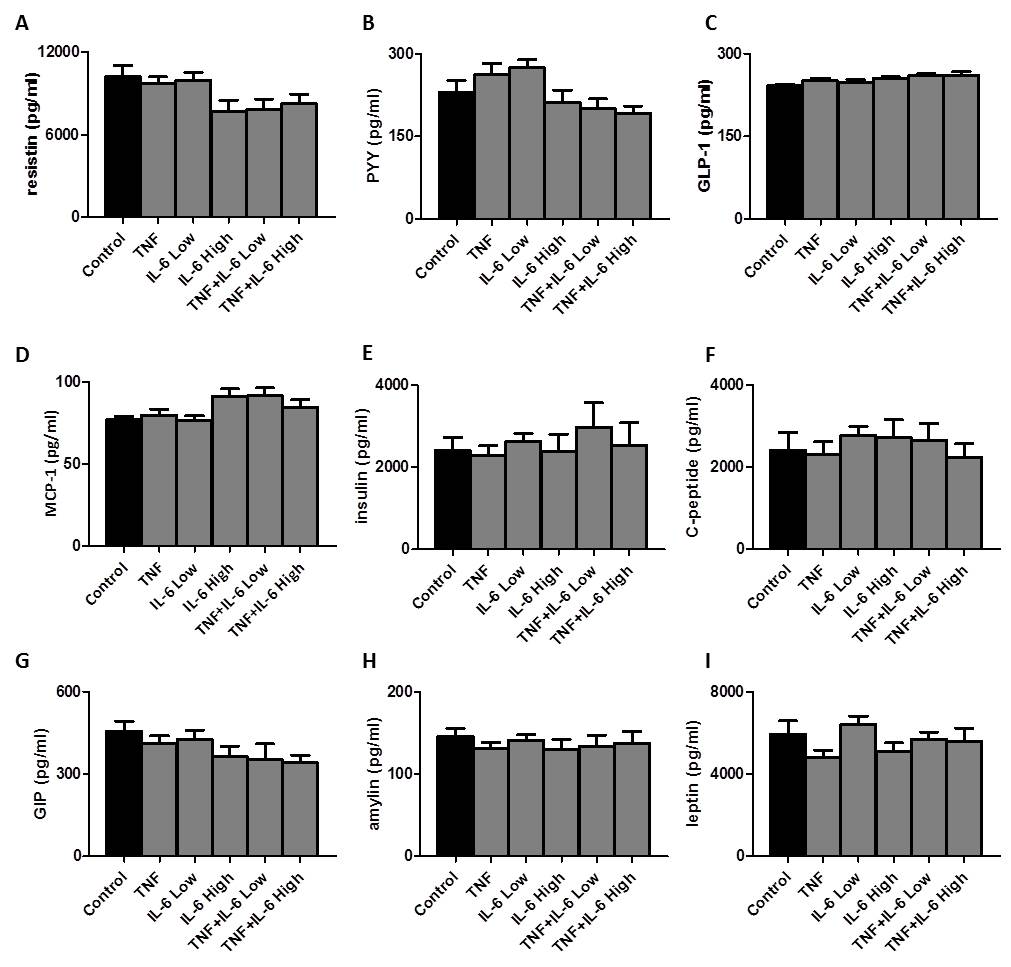

Supplement: Supplementary file 1 — 10.1186/s12868-016-0260-0 Plasma levels of gut hormones. Effect of ipinjection with TNFα, IL-6 or both on A) Resistin, B) Peptide YY, C) Glucagon–like peptide (GLP-1), D) MCP-1, E) Insulin, F) C-peptide, G) Gastric inhibitoryprotein (GIP), H) Amylin, I) Leptin. [file 12868_2016_260_MOESM1_ESM.jpg]
